# CRY1 Lysine 151 Regulates Circadian Rhythms Through Ubiquitination-Independent Protein Interactions

**DOI:** 10.3390/ijms26167962

**Published:** 2025-08-18

**Authors:** Jiawen Peng, Na Liu, Yixuan Ren, Jiahui Wang, Yanxia Jin, Xianping Wang, Weidong Wang, Jicheng Pan

**Affiliations:** Hubei Key Laboratory of Edible Wild Plants Conservation and Utilization, College of Life Sciences, Hubei Normal University, Huangshi 435002, China; 15172030217@163.com (J.P.); renyixuan@stu.hbnu.edu.cn (Y.R.); wangjiahui@stu.hbnu.edu.cn (J.W.); jinyanxia@hbnu.edu.cn (Y.J.); xianpingwang@hbnu.edu.cn (X.W.); wangweidong@hbnu.edu.cn (W.W.)

**Keywords:** cryptochrome 1, circadian clock, ubiquitination-independent, protein interactions

## Abstract

Mammalian circadian rhythms, governing ~24 h oscillations in behavior, physiology, and hormone levels, are orchestrated by transcriptional–translational feedback loops centered around the core clock protein cryptochrome 1 (CRY1). While CRY1 ubiquitination is known to regulate clock function, the roles of specific ubiquitination sites remain unclear. Here, we identify lysine 151 (K151) as a critical residue modulating the circadian period through non-canonical mechanisms. Using site-directed mutagenesis, we generated CRY1-K151Q/R mutants mimicking constitutive deubiquitination. Circadian rescue assays in Cry1/2-deficient cells revealed period shortening (K151Q: −2.25 h; K151R: −1.4 h; n = 3, *p* < 0.01, Student’s *t*-test), demonstrating K151’s functional importance. Despite normal nuclear localization kinetics, K151Q/R mutants exhibited reduced transcriptional repression in luciferase assays, a weakened interaction with BMAL1 by the luciferase complementation assay, and enhanced binding to E3 ligase FBXL12 (but not FBXL3) while showing more stability than wild-type CRY1. Notably, the absence of ubiquitination-linked degradation or altered FBXL3 engagement suggests a ubiquitination-independent mechanism. We propose that CRY1-K151 serves as a structural hub fine-tuning circadian periodicity by modulating core clock protein interactions rather than through traditional ubiquitin-mediated turnover. These findings redefine the mechanistic landscape of post-translational clock regulation and offer new therapeutic avenues for circadian disorders.

## 1. Introduction

The circadian clock orchestrates ~24 h oscillations in behavior, physiology, and hormone levels, representing a fundamental adaptation to environmental cycles [1]. Disruptions to this system are clinically significant, correlating with metabolic disorders [2], neurodegeneration [3], psychiatric disorders [4], and sleep pathologies [5]. Genetic variations have been identified that lead to familial advanced sleep phase (FASP) [5,6,7,8,9,10], delayed sleep phase disorders (FDSP) [11,12], and natural short sleep [6,13,14]. At the molecular level, the transcription–translation feedback loop (TTFL) generates and sustains circadian rhythms under constant conditions [15]. Substantial evidence indicates that perturbing core clock genes encoding transcriptional regulators disrupts rhythmicity, causing period alterations or arrhythmicity [16].

In mammals, the core oscillator comprises the repressor complex PER/CRY and the activator heterodimer CLOCK/BMAL1 [17]. As bHLH-PAS transcription factors, CLOCK and BMAL1 dimerize to drive the transcription of E-box (CACGTG) and E’-box (CACGTT)-containing genes, including Per and Cry. Following translation, PER and CRY proteins accumulate, heterodimerize, translocate to the nucleus, and repress CLOCK/BMAL1-mediated transcription. This autoinhibitory feedback downregulates Per and Cry expression. As repressor complexes subsequently degrade below a critical threshold, CLOCK/BMAL1 activity rebounds, initiating a new ~24 h cycle [18].

Notably, CRY plays a non-redundant role: it binds CLOCK/BMAL1 directly to inhibit transcription independently of PER, while PER requires CRY to stably associate with CLOCK-BMAL1-E-box complexes and exert repression [19]. The PER-CRY complex recruits casein kinase 1δ/ε (CK1δ/ε), promoting CLOCK phosphorylation and the subsequent dissociation of CLOCK/BMAL1 from DNA [20]. Mammals express two CRY homologs with distinct period-modulating functions, while CRY1/CRY2 double-knockout mice and cells become arrhythmic [21,22]. This genetic divergence makes the Cry1-/-Cry2-/- mouse embryonic fibroblast cells (DKO cells) an ideal system for identifying functional CRY1 residues via rhythm rescue assays [16,23].

Although the TTFL constitutes the primary circadian mechanism, precise and stable rhythms require additional regulatory layers, including post-translational modifications (PTMs) [24,25]. Recent studies reveal multiple phosphorylation and ubiquitination sites on CRY1 proteins [16,26]. We previously showed that the phosphomimic mutation of CRY1 can alter the circadian period, presumably by enhancing, weakening, or lacking repression capacity in the feedback loop [16]. Modifications at specific residues alter CRY1 stability, subcellular localization, and activity, thereby fine-tuning circadian parameters [27]. CRY1 degradation is predominantly mediated by the SCF-FBXL3 ubiquitin ligase complex (comprising FBXL3, SKP1, and CULLIN1) [26]. Its homolog FBXL21 exhibits dual spatiotemporal roles: it protects nuclear CRY1 from FBXL3-mediated degradation while it promotes CRY1 degradation in the cytoplasm [28]. Among CRY1’s 31 lysines, 11 are predicted ubiquitination sites [26], yet K151 represents a notable exception: structural analyses localize it near the C-terminal domain essential for BMAL1 binding [29], while the predicted ubiquitination is lacking [26]. We hypothesized that this strategically positioned residue may fine-tune clock function through non-degradative mechanisms. Supporting this notion, phosphomimetic CRY1 mutations alter the circadian period by modulating the repression capacity [16]. Here, we investigate the functional significance of CRY1 lysine 151 (K151), which showed that K151 mutations shorten the circadian period in rescue assays, surprisingly through ubiquitination-independent mechanisms involving altered interactions with core clock proteins rather than changes in protein stability.

## 2. Results

### 2.1. Impact of CRY1-K151 on Circadian Periodicity

**Figure 1:**
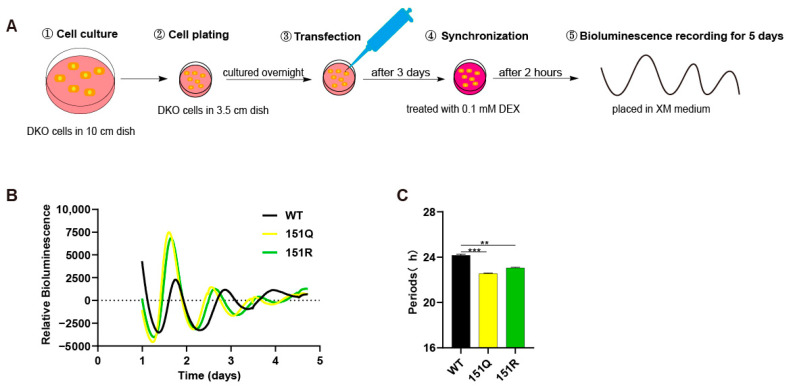
The CRY1-K151Q/R mutant disrupts circadian rhythmicity. (**A**) Schematic diagram of kinetic bioluminescence recording assay. (**B**) Circadian rhythm analysis of P(Per2)-luc reporter activity in DKO cells expressing WT or mutant mCRY1 (baseline subtracted). Cells were co-transfected with mCRY1 expression vectors and the P(Per2)-luc reporter construct. Following a 3-day transfection period, cells were synchronized with dexamethasone treatment and subsequently monitored for bioluminescence over 5–6 days in luciferin-containing medium. Rhythmic data were processed using LumiCycle Analysis software. (**C**) Quantitative comparison of circadian period lengths in cells expressing WT versus mutant mCRY1 constructs. Data represent mean ± SEM (n = 3 independent experiments; ** *p* < 0.01, *** *p* < 0.001, Student’s *t*-test).

Post-translational modifications (PTMs) of CRY1 are critical for mammalian circadian clock function. However, the roles of specific ubiquitinated or acetylated lysine residues—and their acute effects on circadian rhythms—remain poorly characterized. To investigate the functional significance of CRY1 lysine 151 (K151), we leveraged a circadian rescue assay in Cry1-/-Cry2-/- (DKO) cells, wherein transfection of the native-promoter-driven CRY1 construct [P(Cry1)-CRY1] restores rhythmicity, as previously described [16]. We hypothesized that K151 undergoes regulatory ubiquitination. To test this, we generated deubiquitination-mimetic mutants (K151R and K151Q) via site-directed mutagenesis, substituting lysine (K) with arginine (R) or glutamine (Q). These mutants (or wild-type [WT] CRY1) were co-transfected with a Per2-promoter-driven luciferase reporter (containing E-box elements) into DKO cells (Figure 1A,B). The results showed that wild-type (WT) CRY1 rescued circadian rhythms within a period of ~24 h (Figure 1C), which was consistent with our previous study [16]. Surprisingly, both K151 mutants markedly shortened the circadian period: K151Q: −2.25 h (n = 3, *** *p* < 0.001 vs. WT, Student’s *t*-test); K151R: −1.4 h (n = 3,** *p* < 0.01 vs. WT, Student’s *t*-test) (Figure 1C). These results establish K151 as a key residue governing circadian periodicity in mammals.

### 2.2. CRY1-K151 Mutations Delay Initial Protein Degradation Without Altering Proteasome Dependence

**Figure 2:**
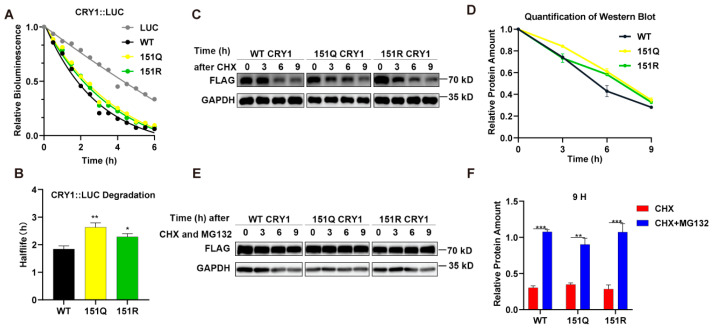
The CRY1-K151Q/R mutant delays initial protein degradation without altering proteasome dependence. (**A**) Luciferase activity of CRY1 fusion proteins. HEK293T cells expressing LUC-mCRY1 (wild-type or mutant) or pcDNA3.1-LUC (control) were treated with cycloheximide (CHX) and luciferin substrate, followed by bioluminescence measurement at 30 min intervals. (**B**) Protein stability analysis of CRY1::LUC fusions. Half-lives were calculated using mono-exponential decay curves from CRY1::LUC degradation profiles. Data represent mean ± SEM from three independent transfections in triplicate samples (n = 9; * *p* < 0.05, ** *p* < 0.01 by Student’s *t*-test). (**C**) Western blot analysis of CRY1 degradation kinetics. HEK293T cells transfected with pCMV-mCRY1 constructs were treated with CHX (100 μg/mL), with protein lysates collected at 0/3/6/9 h post-treatment. Shown are time-course expression profiles of wild-type CRY1 and K151Q/R mutant. (**D**) Quantitative densitometry of K151Q/R mutant degradation. Band intensities were normalized to initial values (0 h) using ImageJ software (version 1.53e) and expressed as percentage remaining. The protein levels at time 0 in each genotype were normalized to 1.0. Error bars represent SEM (n = 3 independent experiments). Two-way ANOVA shows significant statistical differences between WT and K151Q (or K151R) at 6 h (*p* < 0.01). (**E**) Proteasome-dependent degradation assay. Western blot analysis of HEK293T cells co-treated with CHX (100 μg/mL) and proteasome inhibitor MG132 (20 μM) at indicated time points (0, 3, 6, and 9 h) following transfection with pCMV-mCRY1 constructs. (**F**) Quantitative stability analysis after MG132 treatment. The protein levels of wild-type CRY1 and the K151Q/R mutant were analyzed after 9 h of MG132 (20 μM) treatment. Data represent mean ± SEM from three independent transfections (n = 3, ** *p* < 0.01, *** *p* < 0.001 by Student’s *t*-test).

Given the established role of ubiquitination in regulating core clock protein stability, we examined whether deubiquitination-mimetic mutations at CRY1 lysine 151 (K151R/Q) alter the CRY1 half-life (*t*_1/2_), as described in Ref. [30]. We monitored the degradation kinetics of C-terminal luciferase-tagged CRY1 variants (CRY1::LUC) in HEK293T cells following protein synthesis inhibition with cycloheximide (CHX). The luciferase activity was monitored in living cells within 6 h to determine the *t*_1/2_ of the proteins. The results revealed that while all CRY1::LUC fusions degraded faster than luciferase alone (Figure 2A), the K151Q and K151R mutants exhibited prolonged half-lives compared to WT CRY1 (** *p* < 0.01 and * *p* < 0.05, respectively, n = 3, Student’s *t*-test, Figure 2B). We further confirmed this stability phenotype using Flag-tagged constructs. After the transfection of HEK293T cells with Flag-tagged CRY1 (WT or mutant) plasmids, cells were treated with CHX and harvested at the 3rd, 6th, and 9th h. Western blot analysis of CHX-treated samples demonstrated the enhanced stability of both mutants at the 6 h time point relative to WT (n = 3, *p* < 0.01 by two-way ANOVA), though protein levels converged to comparable amounts by 9 h (Figure 2C,D).

To determine whether degradation involved proteasomal pathways, we treated transfected cells with the proteasome inhibitor MG132. This intervention completely blocked the degradation of both WT and mutant CRY1 proteins (n = 3, ** *p* < 0.01, *** *p* < 0.001 by Student’s *t*-test, Figure 2E,F). Crucially, the degradation kinetics remained indistinguishable between genotypes in the absence of MG132 (Figure 2F), demonstrating that proteasome-dependent turnover occurs independently of K151 modification status. Collectively, these data indicate that while K151 mutations transiently stabilize CRY1, they do not alter its fundamental dependence on proteasomal degradation.

### 2.3. K151 Mutations Specifically Enhance CRY1’s Interaction with FBXL21

**Figure 3:**
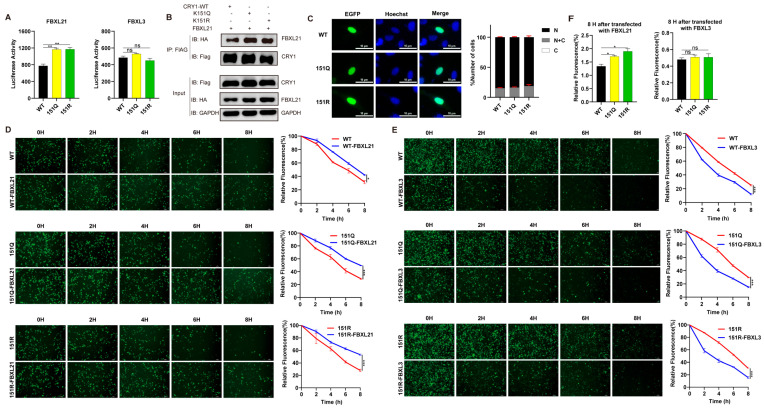
The CRY1-K151Q/R mutation enhances the FBXL21 interaction. (**A**) Quantitative analysis of FBXL21 (or FBXL3) interactions for wild-type and K151Q/R mutant CRY1. Split-luciferase complementation assays revealed differential interactions between mCRY1 (wild-type/mutant) and E3 ligase. HEK293T cells were co-transfected with N-terminal luciferase-tagged CRY1 (wild-type or mutant) and C-terminal luciferase-tagged FBXL21 or FBXL3. Data represent mean ± SEM from three independent transfections in triplicate samples (n = 9, ** *p* < 0.01 by Student’s *t*-test). (**B**) Detection of FLAG-CRY1 (WT or mutants) interaction with HA-FBXL21 in HEK293T cells. The expression constructs were transfected into HEK293T cells as indicated (+). Representative blots from 2 independent experiments are shown. (**C**) Representative images of CRY1 (wild-type and K151Q/R mutant) localization at 20× magnification (left panel). Statistical analysis of CRY1 nucleo-cytoplasmic distribution (right panel). Percentages of cells showing GFP fluorescence in nucleus (N), cytoplasm (C), or both (N + C) are shown. Three distinct regions were analyzed per experiment, with experiments repeated three times to determine the ratio of subcellular localization patterns relative to total transfected cells. (**D**) Representative fluorescence images of FBXL21-dependent degradation of GFP-CRY1 (wild-type or K151Q/R mutant) in HEK293T cells at indicated time points (0, 2, 4, 6, and 8 h) post-CHX treatment. CRY1 protein stability was quantified by normalizing GFP fluorescence intensity to t = 0 h using ImageJ. Qualification data represent mean ± SEM for expression of CRY1 (WT or mutant) alone, or plus with FBXL21 (n = 3 independent experiments; * *p* < 0.05, *** *p* < 0.001 by two-way ANOVA). (**E**) Representative fluorescence images of FBXL3-dependent degradation of GFP-CRY1 (wild-type or K151Q/R mutant) in HEK293T cells at indicated time points (0, 2, 4, 6, and 8 h) post-CHX treatment. CRY1 protein stability was quantified by normalizing GFP fluorescence intensity to t = 0 h using ImageJ analysis. Qualification data represent mean ± SEM for expression of CRY1 (WT or mutant) alone, or plus with FBXL3 (n = 3 independent experiments; *** *p* < 0.001 by two-way ANOVA). (**F**) Quantitative analysis of FBXL21- or FBXL3-mediated effects on protein stability of CRY1 (wild-type and K151Q/R mutants) at the 8 h time point. The relative CRY1 protein levels (both wild-type and mutants) at 8 h post-transfection with FBXL21 or FBXL3 were normalized to pre-transfection baseline levels to assess their differential impacts on CRY1 stability. Data represent mean ± SEM from three independent transfections (n = 3, * *p* < 0.05 by Student’s *t*-test).

Building upon the distinct regulatory roles of SCF ubiquitin ligase complexes—where SCF-FBXL3 mediates CRY degradation while its paralog FBXL21 stabilizes nuclear CRY—we investigated whether K151 mutations alter CRY1’s engagement with these E3 ligases. To achieve this, WT or CRY1 mutants and FBXL3 (or FBXL21) were co-expressed as fusion proteins with N- and C-terminal luciferase fragments in HEK293T cells. We observed that K151Q and K151R mutants maintained wild-type binding affinity for FBXL3 but exhibited a stronger interaction with FBXL21 (n = 9, ** *p* < 0.01 by Student’s *t*-test, Figure 3A), which was confirmed with the co-immunoprecipitation of CRY1-WT and K151Q/R with FBXL21 proteins (Figure 3B). Given FBXL21’s compartment-specific functions, we examined whether K151 mutations affect CRY1 localization. Fluorescence imaging of GFP-tagged CRY1 variants in U2OS cells revealed comparable nuclear enrichment across all genotypes (Figure 3C), indicating that the subcellular distribution remains unaltered by K151 substitutions. This conserved nuclear retention, coupled with enhanced FBXL21 binding (Figure 2B), provides a mechanistic rationale for the observed stabilization of K151 mutants.

To functionally validate these interactions, we tracked the degradation kinetics of GFP-CRY1 variants co-expressed with FBXL3 or FBXL21 in CHX-treated cells. Time-lapse imaging demonstrated three key outcomes: First, FBXL21 exerted its expected stabilizing effect on all CRY1 forms (n = 3, * *p* < 0.05, *** *p* < 0.001 by two-way ANOVA, Figure 3D). Second, FBXL3 comparably accelerated the degradation of wild-type and mutant CRY1 (n = 3, *** *p* < 0.001 by Two-way ANOVA, Figure 3E). Third, K151 mutants displayed substantially prolonged half-lives relative to wild-type, specifically when co-expressed with FBXL21 (n = 3, * *p* < 0.05 by Student’s *t*-test, Figure 3F). Crucially, the degradation kinetics mediated by FBXL3 remained unaltered by K151 mutations. These data collectively establish that K151 modulates CRY1 stability through preferential engagement with the stabilizing factor FBXL21 rather than through altered ubiquitination by the canonical SCF-FBXL3 degradation machinery.

### 2.4. K151 Mutations Impair CRY1 Transcriptional Repression via Disrupted Core Clock Protein Interactions

**Figure 4:**
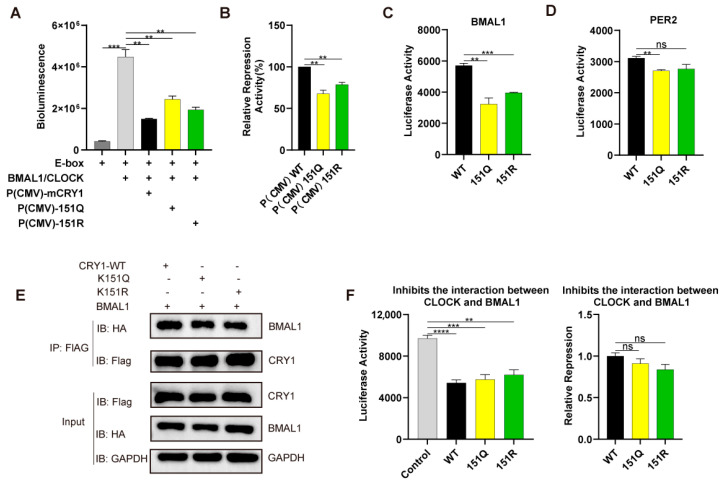
The CRY1-K151Q/R mutation impairs its transcriptional repression capability. (**A**) Impact of CRY1 mutations on transcriptional repression activity. Schematic of the reporter assay system in HEK293 cells co-transfected with BMAL1 (10 ng), CLOCK (15 ng), Per2-luc reporter (10 ng), and either WT or mutant mCRY1 (5 ng). After 24 h, cells were harvested for luciferase activity measurement in luciferin-containing medium. Bar graphs show the mean ± SEM from three independent experiments (n = 3; ** *p* < 0.01, *** *p* < 0.001 by Student’s *t*-test). (**B**) Quantitative analysis of CRY1-mediated transcriptional repression. Luminescence values were normalized to the control condition (transfected with BMAL1:CLOCK and with WT, set as 100%). Results are mean ± SEM for 3 independent experiments (n = 3; ** *p* < 0.01 by Student’s *t*-test). (**C**,**D**) Interaction profiles between (**C**) BMAL1 and (**D**) PER2 with CRY1 (wild-type or mutant). Split-luciferase complementation assays revealed differential interactions between mCRY1 (wild-type/mutant) and core clock components. HEK293T cells were co-transfected with N-terminal luciferase-tagged CRY1 (wild-type or mutant) and C-terminal luciferase-tagged BMAL1 or PER2. Bar graphs show the mean ± SEM from three independent experiments (n = 3; ** *p* < 0.01, *** *p* < 0.001 by Student’s *t*-test). (**E**) Detection of FLAG-CRY1 (WT or mutants) interaction with HA-BMAL1 in HEK293T cells. The expression constructs were transfected into HEK293T cells as indicated (+). Representative blots from 2 independent experiments are shown. (**F**) Functional modulation of BMAL1-CLOCK interaction by CRY1 mutations. (Right) Luciferase complementation assays were performed in HEK293T cells co-expressing BMAL1 and CLOCK (fused to N-terminal and C-terminal luciferase fragments, respectively) with wild-type or mutant CRY1. (Left) BMAL1-CLOCK interaction was normalized to vector control (set as 1) and expressed as fold change. Data represent mean ± SEM from three independent experiments (n = 3; ** *p* < 0.01, *** *p* < 0.001, **** *p* < 0.0001 by Student’s *t*-test).

To assess the functional consequences of K151 mutations on molecular clock regulation, we first measured CRY1’s transcriptional repression capacity using E-box-driven luciferase assays in HEK293T cells. The co-expression of CLOCK/BMAL1 activated luciferase expression, while wild-type (WT) CRY1 under CMV promoter control [P(CMV)] suppressed this activity to ~20% of the baseline (Figure 4A). Paradoxically, despite their enhanced protein stability (Figure 2B), both K151R and K151Q mutants exhibited substantially attenuated repression (n = 3; ** *p* < 0.01 by Student’s *t*-test), approximately 78% and 64% of that of the wild type (Figure 4A,B).

Given CRY1’s established role in inhibiting CLOCK/BMAL1-DNA binding through direct interactions with BMAL1 [31], we hypothesized that K151 mutations compromise these core clock protein engagements. Luciferase complementation assays confirmed substantially weakened binding affinity between K151Q/R mutants and BMAL1, and the binding rate was approximately 50–60% of the wild type (n = 3; ** *p* < 0.01, *** *p* < 0.001 by Student’s *t*-test, Figure 4C). In addition, the binding affinity between K151Q/R mutants and PER2 was approximately 87% of the wild type, while there was no statistically significant reduction in PER2 associated with K151R (Figure 4D). We further detected the interaction between CRY1 (WT or mutant) and BMAL1 through the co-immunoprecipitation assay (Figure 4E), the results of which displayed a similar binding affinity, as shown in Figure 4D. Crucially, when testing the effects on the CLOCK-BMAL1 heterodimer interface, both WT and mutant CRY1 comparably reduced CLOCK-BMAL1 binding, indicating the preserved disruption of activator complex assembly (n = 3; ** *p* < 0.01, *** *p* < 0.001, **** *p* < 0.0001 by Student’s *t*-test, Figure 4F). These collective findings demonstrate that residue K151 governs circadian periodicity primarily by modulating CRY1’s interactions with transcriptional regulators BMAL1, rather than through altered heterodimer dissociation.

## 3. Discussion

Based on our working hypothesis that CRY1-K151 regulates circadian periodicity through ubiquitination-independent mechanisms, the results of this study provide consistent support and thus confirm its validity. This study elucidates a non-canonical regulatory mechanism through which CRY1 lysine 151 governs circadian periodicity, challenging the prevailing paradigm that ubiquitination-dependent degradation primarily dictates clock protein function.

While extensive research has established SCF-FBXL3-mediated ubiquitination as the dominant pathway for CRY1 turnover [26,32], our data demonstrate that K151 mutations accelerate circadian rhythms without altering proteasomal degradation kinetics (Figure 1 and Figure 2). This phenomenon aligns with emerging evidence that certain CRY1 residues, such as S158 and T249, regulate circadian function through stability-independent mechanisms [16], yet it contrasts sharply with the period-lengthening effects observed in the CRY1’s ubiquitination reduced cells [33].

The resolution to this paradox lies in K151’s role as a structural modulator of protein interactions. Our findings reveal that K151Q/R substitutions enhance binding to the nuclear stabilizing factor FBXL21 without affecting FBXL3 engagement (Figure 3A), echoing FBXL21’s compartment-specific protection mechanisms [28]. The nuclear retention of mutant CRY1 (Figure 3B) further supports spatial regulation through FBXL21 shielding [26], as well as prolonged half-lives when co-expressed with FBXL21 (Figure 3C,E). Interestingly, we found that the K151Q/R mutants diminished transcriptional repression (Figure 4A,B) despite increased protein half-life. Structurally, these observations find support in crystal structure analyses localizing K151 near CRY1’s C-terminal fragment—a domain critical for BMAL1 binding [31]. The higher affinity of full-length mCRY1 (vs. C-terminal fragments) for mBMAL1 [29] suggests that N-terminal regions (including K151) allosterically regulate CRY1 interactions. We propose that K151 mutations induce allosteric perturbations that weaken the interaction with BMAL1 while favoring FBXL21 binding, creating a spatial regulatory paradigm where nuclear compartmentalization dictates functional outcomes. Indeed, luciferase complementation and CO-IP assays confirmed the weakened interaction between K151 mutations and BMAL1 (Figure 4C,E). In addition, reserve CRY1’s capacity to dissociate CLOCK-BMAL1 dimers (Figure 4E) confirmed the functional specificity in repressor complex assembly. These findings demonstrate that residue K151 governs circadian periodicity primarily by modulating CRY1’s interactions with FBXL21 and BMAL1.

Beyond mechanistic insights, our work redefines the functional landscape of CRY1 post-translational modifications. Notably, among CRY1’s 31 lysines, K151 is unique in both lacking predicted ubiquitination [26] and operating through ubiquitination-independent mechanisms—a rare paradigm with significant pathophysiological implications. The short-period circadian phenotype induced by CRY1-K151 mutations (Figure 1B) phenocopies the core mechanistic defect underlying familial advanced sleep phase disorder (FASPD)—a condition causally linked to CRY2 mutations in humans [10]. This functional conservation strengthens the pathophysiological relevance of period-altering CRY1 variants in human chronic disorders. While CRY degradation is classically attributed to ubiquitin–proteasome pathways, emerging evidence indicates alternative regulation through autophagy via LC3 binding [23,34]. Intriguingly, K151 resides within a putative LC3-interacting region (LIR) motif (residues 151–156) [34], raising the possibility of autophagic involvement. Furthermore, CRY1 and CRY2 undergo multifaceted PTM crosstalk—including acetylation and phosphorylation—where modifications at one site often influence others [16,35,36]. Future studies should specifically address whether K151 serves as a switch for autophagic degradation or potential competition with acetylation, phosphorylation, and autophagy signals at adjacent sites and how K151-mediated conformational changes propagate to distal functional domains.

Collectively, these findings establish a ubiquitination-independent axis of circadian control where the targeted perturbation of protein interaction networks—rather than altered degradation—drives period determination, offering new therapeutic strategies for circadian disorders that bypass global protein stability modulation.

## 4. Materials and Methods

### 4.1. Cell Lines and Cell Culture

CRY1/2 double-knockout (DKO) cells were generously provided by the laboratory of Dr. Erquan Zhang at the Beijing Institute of Life Sciences (Beijing, China). The HEK293T (Cat# CL-0005) and U2OS (Cat# CL-0236) cell lines were commercially obtained from Procell Life Science & Technology Co., Ltd. (Wuhan, China). All cell lines were maintained in high-glucose Dulbecco’s Modified Eagle Medium (DMEM; Thermo Fisher Scientific, Beijing, China; Cat# C11995500BT) supplemented with 10% fetal bovine serum (FBS; ExCell Bio, Suzhou, China; Cat# FSP500) and 100 U/mL of penicillin–streptomycin (BIOEXPLORER, Guangzhou, China; Cat# B1351-101) under standard culture conditions (37 °C, 5% CO_2_) [37].

### 4.2. Kinetic Bioluminescence Recording

Real-time circadian reporter assays was performed as previously described [16]. CRY1/2 double-knockout (DKO) cells were plated in 35 mm dishes (NEST, Wuxi, China; Cat# 706001) (3–5 × 10^4^ cells/dish) and cultured overnight. Cells were co-transfected with 1 μg of pGL3-P(Per2)-luc reporter, 50 ng of CRY1 plasmid, and 950 ng of pcDNA3.1 (normalization control) using X-tremeGENE HP DNA Transfection Reagent (Roche, Mannheim, Germany; Cat# 0636623600). Three days after transfection, the cells were treated with 0.1 mM of dexamethasone (DEX; Sigma, Saint Louis, MO, USA, Cat# D4902) for 2 h for synchronization and then switched to XM medium [38] for bioluminescence recording in a LumiCycle (36 °C), as previously described [16].

### 4.3. Subcellular Localization Assay

U2OS cells were seeded at a density of 2–4 × 10^5^ cells per well in a 12-well plate and cultured at 37 °C in a 5% CO_2_ incubator for 24 h. For transfection, 2 μg of GFP-tagged CRY1 plasmid (wild-type or mutant) was mixed with Highgene Transfection Reagent (ABclonal, Wuhan, China; Cat# RM09014) and then added dropwise to the cells. Then, 24 h after transfection, the cells were fixed with 4% paraformaldehyde (PFA, Biosharp, Hefei, China; Cat# BL539A) at room temperature for 15 min. The fixative was then removed and the cells were washed three times with PBS (BIOEXPLORER, Guangzhou, China; Cat# B1139-066). For nuclear staining, 100 μL of Hoechst 33,258 (Biosharp, China; Cat# BL803A) solution was added to each well and incubated at room temperature for 10 min in the dark. The staining solution was discarded, and the cells were washed three times with PBS. Finally, the samples were observed using a Nikon ECLIPSE Ti2 microscope (Nikon, Tokyo, Japan) with 20×/0.45 objectives, and images were processed using ImageJ software (National Institutes of Health, Bethesda, MD, USA) to analyze the subcellular localization of mCRY1.

### 4.4. Split-Luciferase Assay

To interrogate direct protein–protein interactions, we implemented a split-luciferase reporter system based on functional complementation [16,29,39]. The N-terminal luciferase fragment (NLuc) was fused to CRY1 (wild-type or mutant), while the C-terminal fragment (CLuc) was fused to the interactional paternal, including FBXL3, FBXL21, BMAL1, and PER2. Cells were co-transfected with NLuc-mCRY1 and CLuc-fusion constructs using HighGene Transfection Reagent. Then, 24 h post-transfection, the cells were changed to DMEM medium containing 100 μM of D-luciferin Potassium Salt (GoldBio, St. Louis, MO, USA; Cat# LUCK), and then bioluminescence signals were recorded using a SpectraMax i3x microplate reader (Molecular Devices, Shanghai, China) to quantify the protein interactions.

### 4.5. Luciferase Repression Assay

HEK293 cells were seeded in 96-well white plates at 1–3 × 10^4^ cells/well in DMEM supplemented with 10% FBS and transfected using HighGene Transfection Reagent with 6 ng of luciferase reporter, as described previously [16], plus core clock components (5 ng of CRY1 expression plasmid, 10 ng of BMAL1, and 15 ng of CLOCK), adjusting to 200 ng of total DNA/well with pcDNA3.1. Bioluminescence signals were recorded 24 h post-transfection, as described in Section 4.4.

### 4.6. Assessment of Protein Stability Using GFP Fluorescence

To evaluate the protein stability of CRY1, we employed a GFP-based degradation assay. HEK293T cells were transiently transfected with plasmids encoding GFP-tagged CRY1 (wild-type or mutant variants). Then, 24 h post-transfection, the cells were treated with 100 μg/mL of cycloheximide (CHX; MedChem Express, Shanghai, China; Cat# HY-12320) to block new protein synthesis. The fluorescence intensity was monitored at 2 h intervals over an 8 h period using a Nikon ECLIPSE Ti2 inverted microscope equipped with a 10×/0.45 objective lens. All images were acquired under consistent exposure conditions and subsequently quantified using ImageJ software to determine the relative protein degradation rates.

### 4.7. Western Blotting

We used RIPA lysis buffer (Beyotime Biotechnology, Shanghai, China; Cat# P0013B) supplemented with a complete protease inhibitor cocktail (Roche, Shanghai, China; Cat# 4693116001). Protein concentrations were determined by the BCA assay (Thermo Fisher Scientific, Waltham, MA, USA; Cat# 23225), with equal amounts (20 μg/lane) resolved by 10% SDS-PAGE and electro-transferred to PVDF membranes (Millipore, Burlington, MA, USA; Cat# IPVH00010). After blocking with 5% non-fat milk in TBST, membranes were incubated with primary antibodies overnight at 4 °C, followed by appropriate HRP-conjugated secondary antibodies for 1 h at room temperature. Protein bands were visualized by a Vilber FUSION FX7 chemiluminescence imager (Vilber, Eberhardzell, Germany) and quantified using ImageJ software. The following antibodies were used for protein detection: anti-GAPDH (Proteintech Group, Wuhan, China; Cat#60004-1-Ig), anti-FLAG (MBL, Beijing, China; Cat# M185-3L), and anti-HA (Huaxingbio, Beijing, China; Cat# HX1820). The secondary antibodies used include goat anti-mouse IgG-HRP (Proteintech Group, Wuhan, China; Cat# SA00001-1) and goat anti-rabbit IgG-HRP (Huaxingbio, Beijing, China; Cat# HX2031). The Flag-tagged CRY1 plasmids (wild-type or mutant) were transfected into HEK293T cells. Then, 24 h post-transfection, the cells were treated with 100 μg/mL of CHX and 20 μM of MG132 (MedChem Express, Shanghai, China; Cat# HY-13259) to block protein synthesis and degradation, respectively. Cells were harvested at 0, 3, 6, and 9 h after the CHX treatment to assess protein stability over time.

### 4.8. Luciferase Degradation Assay

HEK293T cells were transiently transfected with luciferase-tagged CRY1 (LUC-CRY1) plasmid (wild-type or mutant variants). Then, 24 h post-transfection, protein synthesis was inhibited by treatment with CHX in fresh medium containing 100 µM of D-luciferin. Bioluminescence was monitored at 30 min at 36 °C intervals for 8 h using a SpectraMax i3x microplate reader (Molecular Devices). *t*_1/2_ of protein was calculated via the one-phase exponential decay fitting function in GraphPad PRISM (version 9.00, GraphPad Software, Inc., San Diego, CA, USA) [30].

### 4.9. Co-Immunoprecipitation Assay

HEK293T cells were co-transfected with FlAG-CRY1, HA-tagged FBXL21, or BMAL1 expression plasmids. Then, 48 h post-transfection, the cells were treated with 20 μM of MG132 (proteasome inhibitor) for 6 h prior to harvest. The cells were lysed in ice-cold buffer containing 25 mM of Tris-HCl (pH 7.6), 150 mM of NaCl, 1% NP-40, and 0.5 mM of EDTA, supplemented with a protease inhibitor cocktail. For immunoprecipitation, cell lysates were incubated with anti-FlAG antibody at 4 °C overnight, followed by the addition of 40 μL of protein A/G magnetic beads (Beyotime Biotechnology, Shanghai, China; Cat# P2108) for 3 h at 4 °C. The immunocomplexes were washed five times with lysis buffer before elution in 1× SDS loading buffer. Precipitated proteins were resolved by SDS-PAGE and analyzed via immunoblotting using anti-HA and anti-FlAG antibodies.

### 4.10. Statistical Analyses

In all experiments, unless noted, error bars represent SEM. The numbers of repeats and statistical tests used are indicated in the figure legends. Statistical analyses were performed using GraphPad PRISM (version 9.00, GraphPad Software, Inc., San Diego, CA, USA). Comparisons between two groups were made using Student’s unpaired *t*-test. Half-lives were calculated using a one-phase exponential decay function and then analyzed with Student’s *t*-test. Comparisons between WT and mutants for CHX-chase assay in Section 4.6, Section 4.7 and Section 4.8 were performed using two-way ANOVA. * *p* < 0.05, ** *p* < 0.01, *** *p* < 0.001, **** *p* < 0.0001.

## Data Availability

The additional data supporting the manuscript are available from the corresponding author upon request.

## References

[1] Mortimer T., Smith J.G., Muñoz-Cánoves P., Benitah S.A. (2025). Circadian Clock Communication during Homeostasis and Ageing. Nat. Rev. Mol. Cell Biol..

[2] Schrader L.A., Ronnekleiv-Kelly S.M., Hogenesch J.B., Bradfield C.A., Malecki K.M. (2024). Circadian Disruption, Clock Genes, and Metabolic Health. J. Clin. Investig..

[3] Nassan M., Videnovic A. (2022). Circadian Rhythms in Neurodegenerative Disorders. Nat. Rev. Neurol..

[4] Gao Q., Tang Z., Wang H., Yamazaki M., Jiang J., Fu Y.-H., Ptacek L.J., Zhang L. (2024). Human PERIOD3 Variants Lead to Winter Depression-like Behaviours via Glucocorticoid Signalling. Nat. Metab..

[5] Ashbrook L.H., Krystal A.D., Fu Y.-H., Ptáček L.J. (2020). Genetics of the Human Circadian Clock and Sleep Homeostat. Neuropsychopharmacology.

[6] Jones C.R., Campbell S.S., Zone S.E., Cooper F., DeSano A., Murphy P.J., Jones B., Czajkowski L., Ptácek L.J. (1999). Familial Advanced Sleep-Phase Syndrome: A Short-Period Circadian Rhythm Variant in Humans. Nat. Med..

[7] Toh K.L., Jones C.R., He Y., Eide E.J., Hinz W.A., Virshup D.M., Ptácek L.J., Fu Y.H. (2001). An hPer2 Phosphorylation Site Mutation in Familial Advanced Sleep Phase Syndrome. Science.

[8] Webb J.M., Abderemane-Ali F., Ashbrook L., Ma M., Nibber N., Zou X., Yamazaki M., Wohler E., Sobreira N., Minor D.L. (2025). CACNA1D Is a Circadian Gene and Causes Familial Advanced Sleep Phase. Proc. Natl. Acad. Sci. USA.

[9] Silvestri R., Guarnieri B. (2025). Advanced Sleep Phase Syndrome: Role of Genetics and Aging. Handb. Clin. Neurol..

[10] Hirano A., Shi G., Jones C.R., Lipzen A., Pennacchio L.A., Xu Y., Hallows W.C., McMahon T., Yamazaki M., Ptáček L.J. (2016). A Cryptochrome 2 Mutation Yields Advanced Sleep Phase in Humans. eLife.

[11] Patke A., Murphy P.J., Onat O.E., Krieger A.C., Özçelik T., Campbell S.S., Young M.W. (2017). Mutation of the Human Circadian Clock Gene CRY1 in Familial Delayed Sleep Phase Disorder. Cell.

[12] Lucio-Enríquez K.R., Rubio-Valles M., Ramos-Jiménez A., Pérez-León J.A. (2025). Human Melanopsin (OPN4) Gene Polymorphisms: A Systematic Review. Front. Neurosci..

[13] Webb J.M., Ma M., Yin C., Ptáček L.J., Fu Y.-H. (2022). An Excitatory Peri-Tegmental Reticular Nucleus Circuit for Wake Maintenance. Proc. Natl. Acad. Sci. USA.

[14] Pellegrino R., Kavakli I.H., Goel N., Cardinale C.J., Dinges D.F., Kuna S.T., Maislin G., Van Dongen H.P.A., Tufik S., Hogenesch J.B. (2014). A Novel BHLHE41 Variant Is Associated with Short Sleep and Resistance to Sleep Deprivation in Humans. Sleep.

[15] Zhang E.E., Kay S.A. (2010). Clocks Not Winding down: Unravelling Circadian Networks. Nat. Rev. Mol. Cell Biol..

[16] Liu N., Zhang E.E. (2016). Phosphorylation Regulating the Ratio of Intracellular CRY1 Protein Determines the Circadian Period. Front. Neurol..

[17] Li W., Wang Z., Cao J., Dong Y., Chen Y. (2023). Perfecting the Life Clock: The Journey from PTO to TTFL. Int. J. Mol. Sci..

[18] Rosensweig C., Green C.B. (2020). Periodicity, Repression, and the Molecular Architecture of the Mammalian Circadian Clock. Eur. J. Neurosci..

[19] St John P.C., Hirota T., Kay S.A., Doyle F.J. (2014). Spatiotemporal Separation of PER and CRY Posttranslational Regulation in the Mammalian Circadian Clock. Proc. Natl. Acad. Sci. USA.

[20] Cao X., Yang Y., Selby C.P., Liu Z., Sancar A. (2021). Molecular Mechanism of the Repressive Phase of the Mammalian Circadian Clock. Proc. Natl. Acad. Sci. USA.

[21] Börding T., Abdo A.N., Maier B., Gabriel C., Kramer A. (2019). Generation of Human CRY1 and CRY2 Knockout Cells Using Duplex CRISPR/Cas9 Technology. Front. Physiol..

[22] Mammalian Cry1 and Cry2 Are Essential for Maintenance of Circadian Rhythms | Nature. https://www.nature.com/articles/19323.

[23] Parlak G.C., Baris I., Gul S., Kavakli I.H. (2023). Functional Characterization of the CRY2 Circadian Clock Component Variant p.Ser420Phe Revealed a New Degradation Pathway for CRY2. J. Biol. Chem..

[24] Parnell A.A., De Nobrega A.K., Lyons L.C. (2021). Translating around the Clock: Multi-Level Regulation of Post-Transcriptional Processes by the Circadian Clock. Cell Signal.

[25] Anna G., Kannan N.N. (2021). Post-Transcriptional Modulators and Mediators of the Circadian Clock. Chronobiol. Int..

[26] Yoo S.-H., Mohawk J.A., Siepka S.M., Shan Y., Huh S.K., Hong H.-K., Kornblum I., Kumar V., Koike N., Xu M. (2013). Competing E3 Ubiquitin Ligases Govern Circadian Periodicity by Degradation of CRY in Nucleus and Cytoplasm. Cell.

[27] Takahashi J.S. (2017). Transcriptional Architecture of the Mammalian Circadian Clock. Nat. Rev. Genet..

[28] Hirano A., Yumimoto K., Tsunematsu R., Matsumoto M., Oyama M., Kozuka-Hata H., Nakagawa T., Lanjakornsiripan D., Nakayama K.I., Fukada Y. (2013). FBXL21 Regulates Oscillation of the Circadian Clock through Ubiquitination and Stabilization of Cryptochromes. Cell.

[29] Czarna A., Berndt A., Singh H.R., Grudziecki A., Ladurner A.G., Timinszky G., Kramer A., Wolf E. (2013). Structures of Drosophila Cryptochrome and Mouse Cryptochrome1 Provide Insight into Circadian Function. Cell.

[30] Gul S., Aydin C., Ozcan O., Gurkan B., Surme S., Baris I., Kavakli I.H. (2020). The Arg-293 of Cryptochrome1 Is Responsible for the Allosteric Regulation of CLOCK-CRY1 Binding in Circadian Rhythm. J. Biol. Chem..

[31] Parico G.C.G., Perez I., Fribourgh J.L., Hernandez B.N., Lee H.-W., Partch C.L. (2020). The Human CRY1 Tail Controls Circadian Timing by Regulating Its Association with CLOCK:BMAL1. Proc. Natl. Acad. Sci. USA.

[32] Gabriel C.H., del Olmo M., Rizki Widini A., Roshanbin R., Woyde J., Hamza E., Gutu N.-N., Zehtabian A., Ewers H., Granada A. (2024). Circadian Period Is Compensated for Repressor Protein Turnover Rates in Single Cells. Proc. Natl. Acad. Sci. USA.

[33] Gül Z.M., Aydoğan S., Surme S., Harputluoğlu Efendi S.N., Özcan O., Uyanık E., Baris I., Gul S., Kavakli I.H. (2025). M54 Selectively Stabilizes the Circadian Clock Component of CRY1 and Enhances the Period of Circadian Rhythm at Cellular Level. J. Biol. Chem..

[34] Toledo M., Batista-Gonzalez A., Merheb E., Aoun M.L., Tarabra E., Feng D., Sarparanta J., Merlo P., Botrè F., Schwartz G.J. (2018). Autophagy Regulates the Liver Clock and Glucose Metabolism by Degrading CRY1. Cell Metab..

[35] Xia K., Li S., Yang Y., Shi X., Zhao B., Lv L., Xin Z., Kang J., Ren P., Wu H. (2023). Cryptochrome 2 Acetylation Attenuates Its Antiproliferative Effect in Breast Cancer. Cell Death Dis..

[36] Kim Y.Y., Jang H., Lee G., Jeon Y.G., Sohn J.H., Han J.S., Lee W.T., Park J., Huh J.Y., Nahmgoong H. (2022). Hepatic GSK3β-Dependent CRY1 Degradation Contributes to Diabetic Hyperglycemia. Diabetes.

[37] Liu N., Tian H., Yu Z., Zhao H., Li W., Sang D., Lin K., Cui Y., Liao M., Xu Z. (2022). A Highland-Adaptation Mutation of the Epas1 Protein Increases Its Stability and Disrupts the Circadian Clock in the Plateau Pika. Cell Rep..

[38] Zhang E.E., Liu A.C., Hirota T., Miraglia L.J., Welch G., Pongsawakul P.Y., Liu X., Atwood A., Huss J.W., Janes J. (2009). A Genome-Wide RNAi Screen for Modifiers of the Circadian Clock in Human Cells. Cell.

[39] Wu Y., Zhao H., Zhang E.E., Liu N. (2021). Identification of PCBP1 as a Novel Modulator of Mammalian Circadian Clock. Front. Genet..

